# Urodynamic characteristics of different types of detrusor overactivity in patients with benign prostatic obstruction

**DOI:** 10.3389/fruro.2026.1845544

**Published:** 2026-05-22

**Authors:** Xiao Zeng, Bo Huang, Hong Shen, De-yi Luo, Tao Jin

**Affiliations:** 1 Department of Urology, Institute of Urology, West China Hospital, Sichuan University, Chengdu, Sichuan, China; 2 Department of Urology, Guang’an District People’s Hospital, Guang’an, Sichuan, China

**Keywords:** benign prostatic obstruction, detrusor overactivity, phasic detrusor overactivity, terminal detrusor overactivity, urodynamic study

## Abstract

**Background:**

This study aimed to investigate whether the presence or the absence of detrusor overactivity (DO) in patients with urodynamic-confirmed benign prostatic obstruction (BPO) leads to alterations in urodynamic parameters.

**Methods:**

This study is a retrospective analysis of patients with urodynamic-confirmed BPO [Bladder Outlet Obstruction Index (BOOI) >40]. Patients with a clinical diagnosis of benign prostatic hyperplasia (BPH) or benign prostatic enlargement (BPE) were initially screened, but only those with BOOI > 40 were included. Based on the presence or absence of DO during urodynamics, patients were categorized into two groups: BPO with DO and BPO without DO. Subsequently, the BPO with DO group was further stratified according to DO subtype into BPO with terminal DO (TDO) and BPO with phasic DO (PDO). Differences in the urodynamic parameters and the DO-related indices among these groups were then evaluated. In addition, correlations between the DO-related indicators and the urodynamic parameters within each DO subtype were further examined.

**Results:**

There were 75 patients assigned to the BPO with DO group and 71 to the BPO without DO group. There was a statistically significant difference in the maximum cystometric capacity (MCC) and bladder compliance (BC) between the BPO with DO group and the BPO without DO group: 259.05 ± 44.54 *vs.* 273.13 ± 36.69 (*p* = 0.04) and 158.71 ± 83.86 *vs*. 210.95 ± 82.58 (*p* < 0.001), respectively. The BPO with PDO group comprised 38 patients, while the BPO with TDO cohort included 37 patients. There exists a statistically significant difference between MCC and BC: 291.50 (248.75–301.75) *vs*. 250.00 (210.00–296.00) (*p* = 0.02) and 184.00 (150.50–286.75) *vs*. 103.00 (85.00–141.50) (*p* < 0.001), respectively. Univariate regression analysis revealed a statistically significant negative correlation between the maximum contraction amplitude of DO and BC exclusively within the BPO with TDO cohort (*r*_s_ = −0.34, *p* = 0.03). Multivariable analysis confirmed this independent correlation (*β* = −0.33, 95%CI = −0.59 to −0.07, *p* < 0.05). Age was identified as an independent risk factor for DO (OR = 1.19, *p* < 0.001) and first desire to void (FD) as an independent protective factor (OR = 0.98, *p* < 0.001).

**Conclusion:**

This preliminary study indicates that DO is associated with storage-phase urodynamic alterations in BPO patients, with no notable association with the voiding phase parameters. Multivariable analysis identified age as an independent risk factor for DO and FD as an independent protective factor. TDO is associated with more pronounced storage-phase changes than PDO. In the TDO subgroup, the correlation between BC and DO contraction amplitude was independent of age. However, several other comparisons remain unadjusted; therefore, these findings are exploratory. Future prospective studies with larger samples and age-adjusted designs are needed.

## 1 Introduction

Benign prostatic hyperplasia (BPH) is a common condition among middle-aged and elderly men. Patients with BPH may have prostate enlargement, lower urinary tract symptoms (LUTS), and bladder outlet obstruction (BOO). These clinical manifestations often occur in combination (1). In the early stages, patients primarily exhibit LUTS such as urinary frequency and urgency. As the disease progresses, they may experience difficulty in urination and urinary retention. Among those with BPH, between 15% and 30% of men may have LUTS (2). These symptoms significantly impact patients’ quality of life (QOL) and also increase the socioeconomic burden on the healthcare system (3–5).

It is common for patients with BPH to exhibit symptoms of overactive bladder (OAB) (6). A number of studies have indicated that approximately 45%–50% of patients with BPH complicated by BOO may experience detrusor overactivity (DO), which is also one of the main contributing factors to LUTS. In addition, other research works have reported a prevalence of DO in patients with BPH ranging from 22% to 47%, with one study pointing out that the incidence of DO in their study population can be as high as 60.9% (7–11). Concurrently, some researchers have also studied the occurrence of DO after the relief of BOO by surgical treatment. Research indicates that, among patients with BPH who undergo surgical treatment to relieve obstruction, approximately 20%–60% still experience DO (12–15).

DO can only be diagnosed through urodynamic study (UDS), primarily characterized by spontaneous or provoked involuntary contractions of the detrusor muscle during the bladder filling phase. Typically, based on the morphological features of DO, it can be generally classified into two types: phasic detrusor overactivity (PDO) and terminal detrusor overactivity (TDO) (16, 17). PDO is defined by a characteristic waveform and may or may not lead to urinary incontinence, while TDO is defined as a single involuntary detrusor contraction and typically results in bladder emptying voiding (16). Unfortunately, there is currently a lack of relevant research on these two different types of DO and their role in LUTS in BPH. There have been numerous studies conducted on DO in BPH to date. A study indicates that, in patients with BPH, DO is independently associated with the patient’s age and BOO. In addition, the probability of DO increases with patient age and the BOO grade (11). A number of studies have also confirmed that BOO is a risk factor for DO (18) and that DO may be a good predictor of prognosis in patients with BPH combined with acute urinary retention (19). However, some studies indicate that DO cannot predict LUTS in patients after photoselective vaporization of the prostate (PVP) surgery, with some studies even pointing out that they could not observe a significant statistical relationship between DO and BOO (20, 21).

The subtype classification of DO not only facilitates the assessment of the severity of lower urinary tract dysfunction in patients but also guides the selection of therapeutic approaches and provides prognostic indications for patient outcomes (22). Elderly patients are more susceptible to TDO, and the associated LUTS in patients with TDO are generally more severe than those in patients with PDO (23–25). Anti-muscarinic agents have demonstrated limited efficacy in TDO cases, making beta-3 adrenergic agonists the preferred therapeutic option for this patient subgroup (26). With regard to surgical intervention, patients with TDO tend to have less favorable postoperative outcomes compared with those with PDO (27, 28). Therefore, elucidating the specific DO subtype in patients with BOO combined with DO holds significant value for the accurate assessment of lower urinary tract function, the selection of appropriate treatment modalities, and the prediction of patient prognosis.

The primary objective of this study was to investigate the differences in lower urinary tract function between patients with BPH associated with BOO who present with DO and those without DO. In addition, it seeks to analyze the functional differential characteristics and potential clinical implications of the various DO subtypes in this patient population.

## 2 Materials and methods

### 2.1 Sample selection process

**Figure 1:**
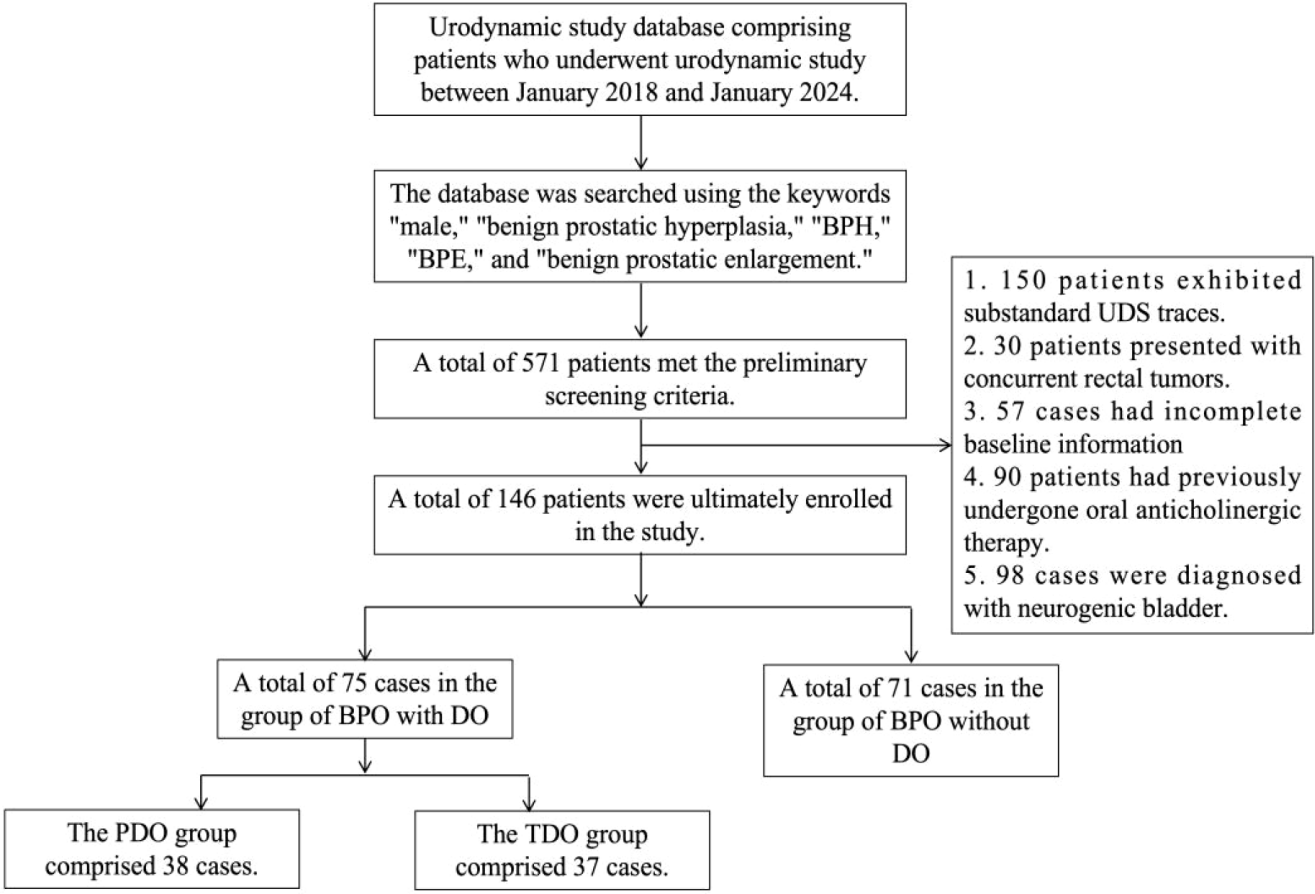
Research flowchart.

This is a retrospective, single-center study based on a retrospective query of the urodynamic database from West China Hospital of Sichuan University, covering the period from January 2018 to January 2024. The cohort did not consist of strictly consecutive patients; rather, it was derived from a consecutive screening of the database according to predefined inclusion and exclusion criteria. The database was searched using the keywords “Male,” “Benign Prostatic Hyperplasia,” “BPH,” “BPE,” and “Benign Prostatic Enlargement.” These keywords were used to identify patients with a clinical diagnosis of BPH/BPE as a starting population. Data extraction and interpretation of urodynamic traces were conducted by two independent urologists, each with over 10 years of experience in urology and urodynamics. For traces with interpretative discrepancies, a third independent researcher provided interpretative suggestions. If the third researcher’s suggestions did not resolve the discrepancies, the trace and related data were excluded from the study. Of note is that formal inter-observer agreement metrics were not calculated for DO subtype classification, which we acknowledge as a limitation (**Figure 1**).

### 2.2 Inclusion and exclusion criteria

The inclusion criteria were as follows: 1) male patients initially identified from the database with a clinical diagnosis of BPH or BPE; 2) patients with normal prostate-specific antigen (PSA) levels or those in whom prostate tumors have been excluded; 3) patients with no history of neurological disorders or pelvic floor surgery; 4) patients with no history of diabetes; 5) patients without urethral strictures; 6) patients with urodynamic traces that are clear and easy to interpret; and 7) urodynamic-confirmed BOO defined by Bladder Outlet Obstruction Index (BOOI) >40 (this is the final and most critical inclusion criterion, which ensures that all enrolled patients have BPO rather than merely clinical BPH/BPE). Patients were subsequently stratified into two subgroups based on the presence or absence of DO observed on UDS: the BPO with DO subgroup and the BPO without DO subgroup. Within the BPO with DO group, patients were further stratified into two subgroups based on the distinct DO subtypes: the BPO with PDO group and the BPO with TDO group.

The exclusion criteria were as follows: 1) radiotherapy of the pelvis; 2) acute urinary tract infection; 3) cognitive dysfunction; 4) coagulation disorders; 5) no uroflow produced during the voiding phase on UDS; 6) patient currently receiving treatment with M-receptor antagonists or β3-adrenoceptor agonists or has undergone intravesical botulinum toxin injections; and 7) patient underwent sacral neuromodulation or tibial nerve modulation therapy.

### 2.3 Urodynamic study process

UDS were conducted in the sitting position using Laborie Triton equipment with air-charged catheters, following the Good Urodynamic Practice (GUP) guidelines (17). All patients were perfused with 37°C sterile normal saline with a filling rate in milliliters per minute of roughly 10% of the largest voided volume (the largest voided volume can be obtained from the patient’s voiding diary).

### 2.4 Urodynamic measurements

The following parameters were determined: 1) maximum cystometric capacity (MCC); 2) bladder compliance (BC); 3) maximum uroflow rate (*Q*_max_); 4) detrusor pressure at maximum uroflow rate (*P*_det_.*Q*_max_); 5) post-void residual volume (PVR); 6) volume at first desire to void (FD); 7) volume at strong desire to void (SD); 8) Bladder Contraction Index (BCI) = *P*_det_.*Q*_max_ + 5.*Q*_max_; and 9) Bladder Outlet Obstruction Index (BOOI) = *P*_det_.*Q*_max_ − 2.*Q*_max_.

### 2.5 Detrusor overactive contraction-related parameters

DO was defined according to the 2002 classification of the International Continence Society (ICS) as spontaneous or provoked (coughing) involuntary detrusor contractions during the bladder filling phase regardless of amplitude. The criteria for determining TDO and PDO also refer to the descriptions provided in this guideline (16). Wet DO was defined as the presence of involuntary detrusor contraction accompanied by observable urinary leakage during the filling phase. Dry DO was defined as the presence of involuntary detrusor contraction without an accompanying urinary leakage.

#### 2.5.1 TDO-related parameters

**Figure 2:**
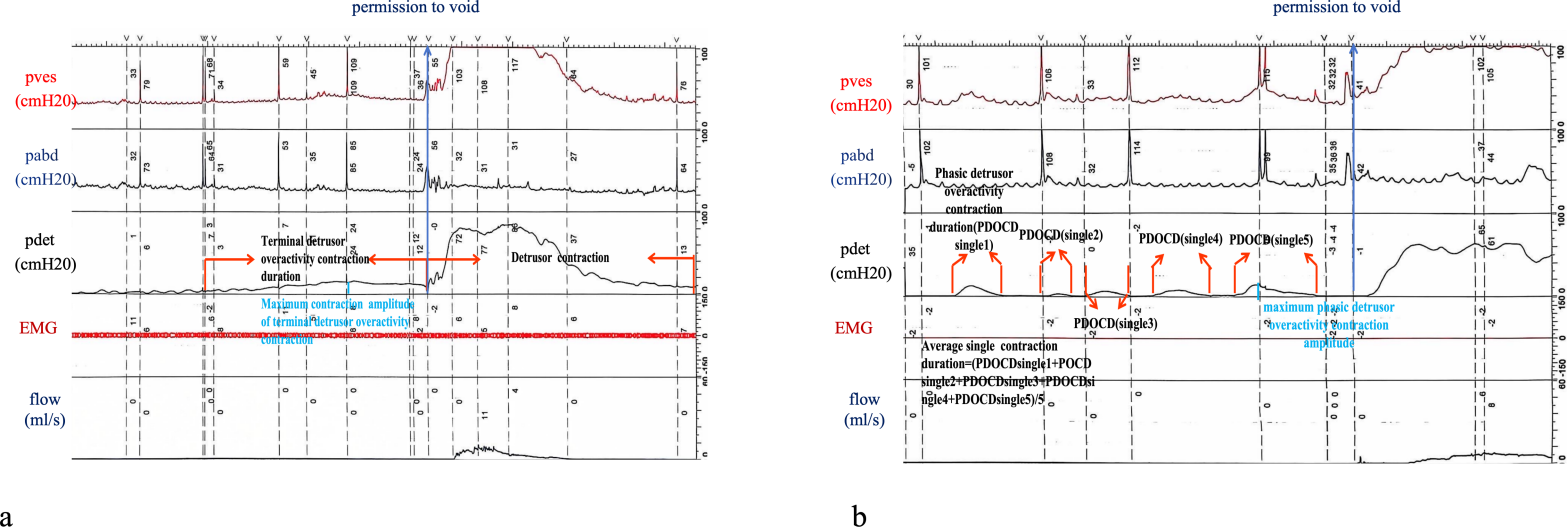
Definition and characteristics of the parameters associated with different types of detrusor overactivity. **(A)** Terminal detrusor overactivity. **(B)** Phasic detrusor overactivity.

**Figure 3:**
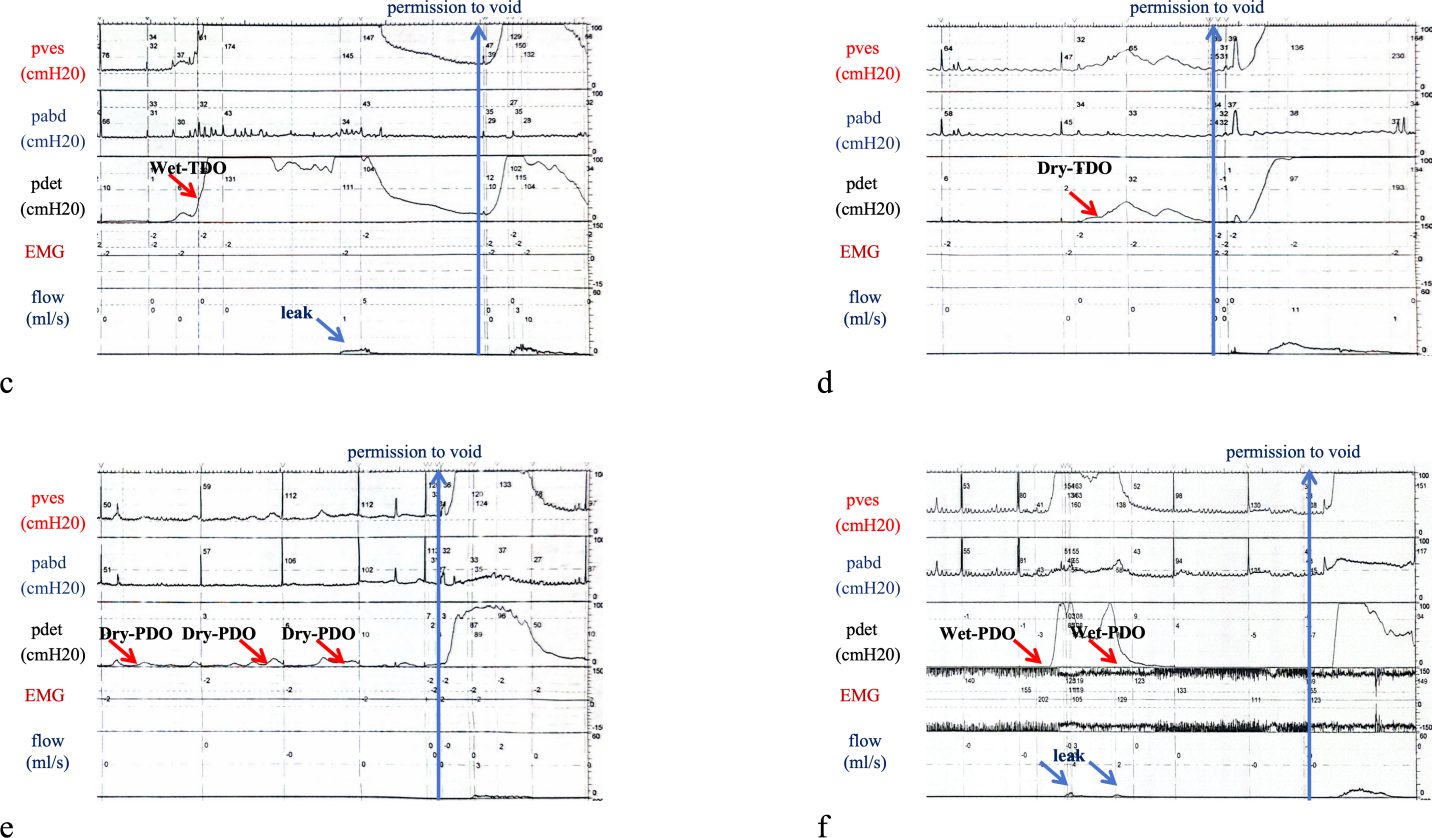
Morphological characteristics of detrusor overactivity in wet *versus* dry conditions. **(C)** Wet terminal detrusor overactivity. **(D)** Dry terminal detrusor overactivity. **(E)** Dry phasic detrusor overactivity. **(F)** Wet phasic detrusor overactivity.

The TDO-related parameters were as follows: 1) maximum amplitude of TDO contraction (in centimeters of the water column, cmH_2_0); 2) TDO contraction duration (in seconds): from the initiation of DO to the completion of the same, the curve returns to the baseline; 3) bladder capacity at the first detection of DO; and 4) bladder capacity at the first detection of TDO: bladder volume at the first occurrence of TDO (**Figures 2A**, **3C, D**).

#### 2.5.2 PDO-related parameters

The PDO-related parameters were as follows: 1) the maximum amplitude among all PDO contraction waveforms (in cmH_2_0); 2) the average single contraction duration: defined as the ratio of the sum of the contraction durations across all PDO waveforms to the number of PDOs (in seconds); and 3) the bladder capacity at the first detection of PDO: the bladder volume at the first occurrence of PDO (**Figures 2B**, **3E, F**).

### 2.6 Medical ethics

This study received approval from the Medical Ethics Committee of the West China Hospital of Sichuan University (no. 2789) and was conducted in strict adherence to the principles outlined in the Declaration of Helsinki. All patients who underwent examination provided informed consent prior to the procedure.

### 2.7 Statistical analysis

Statistical analysis in this study utilized SPSS 21.0, R4.3.2, and GraphPad Prism 8 software. The Shapiro–Wilk test was used to assess normality. For normally distributed and homogeneous numerical data, Student’s *t*-test and analysis of variance were employed to test for intergroup differences, with mean ± standard deviation (SD) used for statistical description. Conversely, for skewed or unequal numerical data distribution, the Mann–Whitney *U* rank-sum test was applied to test for intergroup differences, with *M* (P25–P75) used for the description. For variables following a normal distribution, correlations were assessed using Pearson’s correlation coefficient; for variables that did not meet the normality assumption, Spearman’s rank correlation coefficient was applied. Multivariable logistic regression was used to identify independent risk factors for the occurrence of DO. Linear regression was used to assess whether the correlation between DO contraction amplitude and bladder compliance was independent of confounders. Statistical significance was defined as *p* < 0.05.

## 3 Results

### 3.1 Research data baseline characteristics

After applying rigorous inclusion and exclusion criteria, a total of 146 patients with BPO were ultimately enrolled in the study. Among them, 75 were assigned to the BPO with DO group, with a mean age of 71.37 ± 7.71 years, and 71 to the BPO without DO group, with a mean age of 61.68 ± 7.52 years. The difference in age between the two groups was statistically significant (*p* < 0.001) (**Table 1**).

**Table 1:** Comparative analysis of the urodynamic parameters between the benign prostatic obstruction (BPO) with detrusor overactivity (DO) group and the BPO without DO group.

Index	BPO without DO (*n* = 71)	BPO with DO (*n* = 75)	*p*
Age (years)	61.68 ± 7.52	71.37 ± 7.71	0.00
MCC (ml)	273.13 ± 36.69	259.05 ± 44.54	0.04
FD (ml)	170.44 ± 53.98	140.81 ± 45.56	0.00
SD (ml)	272.00 ± 37.69	256.28 ± 45.88	0.03
BC (ml/cmH_2_0)	210.95 ± 82.58	158.71 ± 83.86	0.00
*Q*_max_ (ml/s)	6.38 ± 2.68	6.40 ± 2.99	0.97
*P*_det_.*Q*_max_	83.63 ± 23.95	84.04 ± 26.24	0.92
BCI	115.54 ± 29.73	116.17 ± 29.65	0.90
BOOI	71.00 ± 23.21	71.37 ± 27.04	0.93
PVR (ml)	115.06 ± 78.05	100.55 ± 76.44	0.26

MCC, maximum bladder cystometry; FD, first desire to void; SD, strong desire to void; BC, bladder compliance; BCI, Bladder Contraction Index; BOOI, Bladder Outlet Obstruction Index; PVR, post-void residual volume; BPH, benign prostatic hyperplasia; BPO, benign prostatic obstruction; DO, detrusor overactivity.

### 3.2 Comparison of the urodynamic parameters between the BPO with DO and BPO without DO groups

There was a statistically significant difference in the urodynamic parameters during the storage phase between the BPO with DO group and the BPO without DO group: MCC = 259.05 ± 44.54 *vs*. 273.13 ± 36.69 (*p* = 0.04); FD = 140.81 ± 45.56 *vs*. 170.44 ± 53.98 (*p* < 0.001); SD = 256.28 ± 45.88 *vs*. 272.00 ± 37.69 (*p* = 0.03); and BC = 158.71 ± 83.86 *vs*. 210.95 ± 82.58 (*p* < 0.001). No statistically significant differences were observed in the voiding phase parameters or the PVR between the two groups: *Q*_max_ = 6.40 ± 2.99 *vs*. 6.38 ± 2.68 (*p* = 0.97); *P*_det_.*Q*_max_ = 84.04 ± 26.24 *vs*. 83.63 ± 23.95 (*p* = 0.92); BCI = 116.17 ± 29.65 *vs*. 115.54 ± 29.73 (*p* = 0.90); BOOI = 71.37 ± 27.04 *vs*. 71.00 ± 23.21 (*p* = 0.93); and PVR = 100.55 ± 76.44 *vs*. 115.06 ± 78.05 (*p* = 0.26) (**Table 1**).

### 3.3 Characteristics among the different subtypes of DO within BPO-DO patients

The BPO with PDO group comprised 38 patients with a mean age of 67.50 (62.00–78.50) years, while the BPO with TDO cohort included 37 patients with a mean age of 72.00 (70.00–76.00) years. No statistically significant difference in age was observed between the two groups (*p* = 0.08). Statistically significant differences were observed in all DO-related parameters between the two groups: maximum amplitude of DO = 13.00 (8.50–32.00) *vs*. 69.00 (32.00–102.00) (*p* < 0.001); contraction duration for DO = 20.00 (15.00–34.50) *vs*. 123.00 (93.00–180.00) (*p* < 0.001); and bladder capacity at the first detection of DO = 155.00 (92.25–189.00) *vs*. 200.00 (155.00–210.00) (*p* < 0.001). There exist statistically significant differences between the MCC, SD, and BC between the two storage-phase urodynamic parameters: 291.50 (248.75–301.75) *vs*. 250.00 (210.00–296.00) (*p* = 0.02); 279.50 (248.75–301.00) *vs*. 248.00 (210.00–296.00) (*p* = 0.03); and 184.00 (150.50–286.75) *vs*. 103.00 (85.00–141.50) (*p* < 0.001), respectively. No statistically significant differences were observed in the voiding phase parameters or the PVR between the two groups (**Table 2**).

**Table 2:** Analysis of the differences in the urodynamic parameters among morphological subgroups following benign prostatic obstruction (BPO) combination with the detrusor overactivity (DO) groups.

Index	BPO with PDO (*n* = 38)	BPO with TDO (*n* = 37)	*p*
Age (years)	67.50 (62.00–78.50)	72.00 (70.00–76.00)	0.08
Maximum amplitude (cmH_2_O)	13.00 (8.50–32.00)	69.00 (32.00–102.00)	0.00
Contraction duration (s)	20.00 (15.00–34.50)	123.00 (93.00–180.00)	0.00
Bladder capacity at first detection of DO (ml)	155.00 (92.25–189.00)	200.00 (155.00–210.00)	0.00
MCC (ml)	291.50 (248.75–301.75)	250.00 (210.00–296.00)	0.02
FD (ml)	136.47 ± 43.98	145.27 ± 47.32	0.41
SD (ml)	279.50 (248.75–301.00)	248.00 (210.00–296.00)	0.03
BC (ml/cmH_2_0)	184.00 (150.50–286.75)	103.00 (85.00–141.50)	0.00
*Q*_max_ (ml/s)	6.92 ± 2.75	5.86 ± 3.16	0.13
*P*_det_.*Q*_max_	76.00 (63.25–94.75)	81.00 (67.00–97.00)	0.54
BCI	111.00 (99.25–138.50)	114.00 (97.00–134.00)	0.91
BOOI	60.00 (48.75–82.75)	68.00 (57.00–82.00)	0.36
PVR (ml)	94.00 (39.75–145.00)	106.00 (47.00–145.00)	0.59

Contraction duration for phasic detrusor overactivity (PDO) is defined as the ratio of the sum of the contraction durations across all PDO waveforms to the number of PDOs.

MCC, maximum bladder cystometry; FD, first desire to void; SD, strong desire to void; BC, bladder compliance; BCI, Bladder Contraction Index; BOOI, Bladder Outlet Obstruction Index; PVR, post-void residual volume; BPH, benign prostatic hyperplasia; BPO, benign prostatic obstruction; DO, detrusor overactivity; PDO, phasic detrusor overactivity; TDO, terminal detrusor overactivity.

### 3.4 Subgroup analysis of the baseline patient characteristics in the BPO with DO cohort stratified by wet *versus* dry DO

Among all patients diagnosed with BPO with DO, 60 cases were identified as dry DO, with a mean age of 70.68 ± 8.09 years, and 15 cases were classified as wet DO, with an average age of 74.13 ± 5.36 years. No statistically significant difference in age was observed between the two groups (*p* = 0.12). Of the DO-related parameters, the maximum amplitude of DO [19.00 (10.00–47.25) *vs*. 104.00 (85.50–135.50), *p* < 0.001] and the contraction duration of DO [36.00 (16.50–95.25) *vs*. 165.00 (104.50–271.50), *p* < 0.001] exhibited statistically significant differences between the two groups, whereas no statistically significant differences were observed for bladder capacity at the first detection of DO [171.50 (127.00–201.00) *vs*. 192.00 (155.00–202.00), *p* = 0.44] between the two groups. During the storage phase, statistically significant differences were observed among the parameters MCC [283.00 (245.50–301.00) *vs*. 223.00 (201.00–255.50), *p* = 0.01], SD [269.50 (233.25–301.00) *vs*. 223.00 (201.00–255.50), *p* = 0.01], and BC [152.00 (113.00–256.50) *vs*. 86.00 (50.46–112.00), *p* < 0.001], whereas no statistically significant differences were detected among the urodynamic indices during the voiding phase (**Table 3**).

**Table 3:** Analysis of the urodynamic parameters among subgroups following classification of benign prostatic obstruction (BPO) patients with dry or wet detrusor overactivity (DO).

Index	BPO with dry DO (*n* = 60)	BPO with wet DO (*n* = 15)	*p*
Age (years)	70.68 ± 8.09	74.13 ± 5.36	0.12
Maximum amplitude (cmH_2_O)	19.00 (10.00–47.25)	104.00 (85.50–135.50)	0.00
Contraction duration (s)	36.00 (16.50–95.25)	165.00 (104.50–271.50)	0.00
Bladder capacity at first detection of DO (ml)	171.50 (127.00–201.00)	192.00(155.00,202.00)	0.44
MCC (ml)	283.00 (245.50–301.00)	223.00 (201.00–255.50)	0.01
FD (ml)	140.48 ± 47.02	142.13 ± 40.69	0.92
SD (ml)	269.50 (233.25–301.00)	223.00 (201.00–255.50)	0.01
BC (ml/cmH_2_0)	152.00 (113.00–256.50)	86.00 (50.46–112.00)	0.00
*Q*_max_ (ml/s)	6.00 (4.00–8.00)	8.00 (4.50–8.50)	0.39
*P*_det_.*Q*_max_	77.50 (69.25–97.50)	74.00 (62.00–92.00)	0.38
BCI	112.00 (99.00–135.50)	112.00 (95.50–134.50)	0.83
BOOI	67.00 (53.00–85.50)	60.00 (47.00–78.00)	0.25
PVR (ml)	101.00 (47.75–145.75)	49.00 (6.00–155.50)	0.17

Contraction duration for phasic detrusor overactivity (PDO) is defined as the ratio of the sum of the contraction durations across all PDO waveforms to the number of PDOs.

MCC, maximum bladder cystometry; FD, first desire to void; SD, strong desire to void; BC, bladder compliance; BCI, Bladder Contraction Index; BOOI, Bladder Outlet Obstruction Index; PVR, post-void residual volume; BPH, benign prostatic hyperplasia; BPO, benign prostatic obstruction; DO, detrusor overactivity.

### 3.5 Correlation analysis between the DO-related indices and the urodynamic parameters across the different DO subgroups within the BPO with DO cohort

Univariate regression analysis revealed a statistically significant negative correlation between the maximum contraction amplitude of the DO and BC exclusively within the BPO with TDO cohort (*r*_s_ = −0.34, *p* = 0.03). No significant associations were observed between other DO-related parameters and the urodynamic measures in the remaining groups. After further eliminating the interference of confounding factors via linear regression, in the BPO combined with TDO group, BC was independently and negatively correlated with the maximum contraction amplitude of DO (*β* = −0.33, 95%CI = −0.59 to −0.07, *p* < 0.05). In the BPO combined with PDO group, after adjusting for age and other covariates, the associations between BC and all DO parameters did not reach statistical significance (all *p* > 0.05) (**Tables 4**, **5**).

**Table 4:** Correlation analysis between the detrusor overactivity (DO)-related indices and the urodynamic parameters among benign prostatic obstruction (BPO) with DO subgroups.

Group	Index	Age	BC	SD	MCC	PVR	*P*_det_.*Q*_max_	FD	BCI	BOOI	*Q*_max_	Value
BPO with PDO	Maximum amplitude (cmH_2_O)	0.31	−0.27	−0.24	−0.16	−0.11	−0.1	−0.1	−0.06	−0.06	−0.05	*r*_s_
		0.06	0.08	0.12	0.34	0.52	0.56	0.57	0.71	0.75	0.77	*p*
	Contraction duration (s)	0.30	−0.30	−0.30	−0.21	−0.09	−0.21	−0.18	−0.16	−0.18	−0.09	*r*_s_
		0.07	0.06	0.07	0.22	0.58	0.22	0.29	0.35	0.28	0.59	*p*
BPO with TDO	Maximum amplitude (cmH_2_O)	0.03^a^	−0.34^b^	−0.26^b^	−0.27^b^	−0.08^b^	0.03^b^	−0.05^a^	0.10^a^	0.00^b^	0.16^a^	*r*_s_/*r*_p_
		0.83	0.03	0.12	0.11	0.64	0.85	0.76	0.55	0.98	0.34	*p*
	Contraction duration (s)	0.12	−0.24	0.25	0.24	0.2	0.07	−0.11	−0.04	0.11	−0.19	*r*_s_
		0.5	0.16	0.14	0.16	0.24	0.69	0.5	0.8	0.51	0.27	*p*

Contraction duration for phasic detrusor overactivity (PDO) is defined as the ratio of the sum of the contraction durations across all PDO waveforms to the number of PDOs. *r*_s_ denotes Spearman’s correlation analysis, while *r*_p_ is Pearson’s correlation analysis.

MCC, maximum bladder cystometry; FD, first desire to void; SD, strong desire to void; BC, bladder compliance; BCI, Bladder Contraction Index; BOOI, Bladder Outlet Obstruction Index; PVR, post-void residual volume; BPH, benign prostatic hyperplasia; BPO, benign prostatic obstruction; DO, detrusor overactivity; PDO, phasic detrusor overactivity; TDO, terminal detrusor overactivity.

a Pearson’s correlation analysis was used.

b Spearman’s correlation analysis was used.

**Table 5:** Correlation analysis between the bladder compliance indicators and the detrusor overactivity (DO)-related parameters among the different DO subgroups with bladder outlet obstruction.

Group	Index	Model 1: *β* (95%CI)	Model 2: *β* (95%CI)	Model 3: *β* (95%CI)
BPO+PDO	Maximum amplitude (cmH_2_O)	−0.12 (−0.24 to −0.00)*	−0.10 (−0.23 to 0.03)	−0.07 (−0.22 to 0.08)
	Contraction duration (s)	−0.13 (−0.25 to −0.00)*	−0.12 (−0.25 to 0.02)	−0.09 (−0.24 to 0.07)
BPO+TDO	Maximum amplitude (cmH_2_O)	−0.29 (−0.51 to −0.06)*	−0.29 (−0.52 to −0.06)*	−0.29 (−0.53 to −0.04)*
	Contraction duration (s)	−0.40 (−1.05 to 0.25)	−0.42 (−1.09 to 0.25)	−0.43 (−1.15 to 0.30)

Model 1, no variables have been adjusted; model 2, the age variable was adjusted for confounding effects; model 3, the effects of multiple variables including age, volume at strong desire to void (SD), post-void residual volume (PVR), first desire to void (FD), Bladder Contraction Index (BCI), and Bladder Outlet Obstruction Index (BOOI) were adjusted for.

BPO, benign prostatic obstruction; DO, detrusor overactivity; PDO, phasic detrusor overactivity; TDO, terminal detrusor overactivity.

*p < 0.05.

### 3.6 Multivariate correlation analysis between the urodynamic parameters and the occurrence of detrusor overactivity

Multivariate logistic regression analysis showed that, for each 1-year increase in age, the risk of DO increased by 19% (OR = 1.19, 95%CI = 1.12–1.28, *p* < 0.001). For each 1-ml increase in FD, the risk of DO decreased by 2% (OR = 0.98, 95%CI = 0.97–0.99, *p* = 0.00). No independent correlation with DO was found for the remaining parameters (i.e., SD, BC, BCI, BOOI, and PVR; all *p* > 0.05) (**Table 6**).

**Table 6:** Multivariate correlation analysis between the urodynamic parameters and the occurrence of detrusor overactivity.

Index	OR (95%CI)	*p*
Age	1.19 (1.12–1.28)	0.00
FD	0.98 (0.97–0.99)	0.00
SD	1.00 (0.99–1.01)	0.93
BC	1.00 (0.99–1.00)	0.38
BCI	1.01 (0.99–1.04)	0.37
BOOI	0.99 (0.96–1.02)	0.57
PVR	1.00 (0.99–1.01)	0.55

FD, first desire to void; SD, strong desire to void; BC, bladder compliance; BCI, Bladder Contraction Index; BOOI, Bladder Outlet Obstruction Index; PVR, post-void residual volume.

## 4 Discussion

As global population aging intensifies, BPH is being increasingly recognized as a significant topic in urology research (29). Previous guidelines from the European Association of Urology (EAU) and the American Urological Association (AUA) did not highly recommend pressure–flow studies (PFS) for the diagnosis of BPH due to limited high-level evidence (1, 5, 30). Furthermore, the optimal timing for surgery in BPH is not explicitly addressed in these guidelines, which primarily outline surgical indications rather than specify when surgery should occur. Current indications include refractory urinary retention, recurrent urinary tract infections, renal insufficiency, bladder stones, and refractory LUTS that do not respond to other treatments (30, 31). Increasingly, researchers are reevaluating the timing of BPH surgery from a functional urology perspective, aiming to promptly intervene in order to prevent disease progression and its associated complications.

A review of previous studies on the efficacy and prognosis of BPH surgery highlights an emerging trend in exploring new urodynamic indicators, particularly focusing on the relationship between DO and BPO. Many studies have demonstrated that patients with DO tend to be older, have a lower MCC, and show higher *P*_det_.*Q*_max_ and BOOI (23, 32, 33). In our study, we similarly observed that the BPO with DO group was significantly older compared with the group without DO (71.37 ± 7.71 *vs*. 61.68 ± 7.52, *p* < 0.001). In addition, we found that the BPO with DO group exhibited a lower MCC (259.05 ± 44.54 *vs*. 273.13 ± 36.69, *p* = 0.04), consistent with prior research findings. Interestingly, we did not find significant statistical differences between the two groups in terms of *Q*_max_, *P*_det_.*Q*_max_, BCI, and BOOI. These findings may indicate that the presence or absence of DO does not significantly impact the functional parameters during the voiding phase in patients with BPO. The lower MCC observed in the BPO with DO group may be attributed to detrusor instability during the storage phase. We further compared the MCC differences between the TDO and PDO groups. Notably, upon further stratification of patients in the BPO with DO group by DO subtype, we observed that the MCC was lower in the BPO with TDO subgroup compared with the BPO with PDO subgroup [250.00 (210.00–296.00) *vs*. 291.50 (248.75–301.75), *p* = 0.02].

According to the ICS definitions, TDO is more likely to result in complete urinary incontinence. This is likely the predominant factor contributing to the reduced MCC observed in the TDO group. Some studies have suggested that the BPO with DO group may exhibit larger *Q*_max_ values (15). However, our analysis failed to yield consistent results, and further subgroup analysis of DO did not reveal significant statistical differences in *Q*_max_ as well. The aforementioned findings indicate that the primary functional discrepancy between BPO patients with DO and those without DO manifests during the storage phase, while no significant functional differences are observed during the voiding phase. Moreover, the principal differences attributable to the various DO subgroups are also predominantly confined to the storage phase, with no statistically significant distinctions identified in terms of voiding function.

In addition to analyzing the differential characteristics of the urodynamic parameters among subgroups, we also examined the differences in the DO-related parameters across these subgroups. The findings from Cubuk et al. indicate that the contraction wave amplitude in patients with TDO is significantly higher than that in PDO cases, a phenomenon that has been substantiated in our study [69.00 (32.00–102.00) *vs*. 13.00 (8.50–32.00), *p* < 0.001]. In our research, we also observed that the contraction duration of TDO was significantly longer than that of PDO [123.00 (93.00–180.00) *vs*. 20.00 (15.00–34.50), *p* < 0.001]. Cubuk suggested that TDO may be associated with a more forceful and abrupt loss of bladder control (23). Concurrently, we observed that the bladder volume at the first occurrence of TDO was significantly greater than that at the first occurrence of PDO [123.00 (93.00–180.00) *vs*. 20.00 (15.00–34.50), *p* < 0.001]. This discrepancy can be attributed to the morphological characteristics of these two types of DO, as TDO typically manifests during the terminal phase of urine storage. We further categorized the BPO with DO group into the dry DO and wet DO subgroups based on the presence or absence of urinary leakage and proceeded with comparative analyses of the DO-related parameters between these subgroups. Our findings indicate that the duration of DO contraction and the maximum contraction amplitude in the wet DO group were both greater than those in the dry DO group. However, due to the significantly smaller sample size in the wet DO group compared with the dry DO group, the current conclusion necessitates further validation through studies with larger sample sizes.

One previous correlational study indicated a positive relationship between the occurrence of DO and BOOI. In addition, MCC is also a factor associated with DO cases. However, this study did not subtype DO. In our study, we divided the DO group into two subgroups, i.e., TDO and PDO, and conducted correlation analyses between the DO-related indices for the different types of DO and the urodynamic parameters. It is noteworthy that a negative correlation between the maximum contractile amplitude of DO and the BC was observed exclusively within the BPO with TDO group (*r*_s_ = −0.34, *p* = 0.03). To further assess whether this association is independent of potential confounders, we performed linear regression analysis. After adjusting for age, MCC, BC, BOOI, and PVR, BC remained independently and negatively correlated with the maximum contraction amplitude of DO in the TDO subgroup (*β* = −0.33, 95%CI = −0.59 to −0.07, *p* < 0.05). In contrast, no significant associations were observed in the PDO subgroup after multivariable adjustment (all *p* > 0.05). Nevertheless, residual confounding due to unmeasured variables cannot be entirely ruled out, and no causal inferences can be drawn from this observational study.

This study has several limitations. Firstly, it is a single-center retrospective analysis, and the cohort did not consist of strictly consecutive patients, which may have limited its generalizability. Secondly, several other urodynamic comparisons remain unadjusted; given the age difference, these findings should be interpreted with caution as exploratory and hypothesis-generating. Thirdly, the DO subtype classification lacked formal inter-observer agreement metrics, limiting reproducibility. Fourthly, subgroup analyses were limited by the small sample sizes, increasing type II error risk; these findings are exploratory. Lastly, future studies with larger samples and prespecified multivariable adjustment are needed to validate all findings.

## 6 Conclusions

This study revealed the characteristics of the different DO types in patients with BPO. DO is associated with storage-phase alterations but not voiding phase parameters. TDO showed worse storage-phase parameters than PDO, with no voiding phase differences.

Multivariable analyses confirmed that, in the TDO subgroup, the BC–DO contraction amplitude correlation is independent of age and identified age as an independent risk factor for DO. However, several unadjusted comparisons should be interpreted with caution given the significant age difference. Future prospective studies with larger samples and age-adjusted designs are needed.

## Data Availability

The raw data supporting the conclusions of this article will be made available by the authors, without undue reservation.
